# BT-CAP: a subcomponent-aware and anatomically constrained data augmentation framework for multi-modal brain tumor MRI segmentation

**DOI:** 10.3389/fradi.2026.1785108

**Published:** 2026-05-12

**Authors:** Amin Tavallaii, Shamim Shah Ghasi

**Affiliations:** 1 Computational Neurosurgery Lab, Department of Neurosurgery, Macquarie University, Sydney, NSW, Australia; 2 Iranian Pediatric Neurosurgery Research Center, Tehran University of Medical Sciences, Tehran, Iran; 3 Department of Neurosurgery, Mashhad University of Medical Sciences, Mashhad, Iran

**Keywords:** brain tumor, data augmentation, deep learning, multimodal MRI, segmentation

## Abstract

**Background:**

Data scarcity and class imbalance remain critical challenges in medical image analysis, particularly for brain tumor MRI segmentation, where subcomponents such as enhancing tumor, non-enhancing tumor, cystic component, and peritumoral edema are underrepresented. Existing augmentation strategies, from classical geometric transforms to GAN-based and diffusion model-based synthesis, either lack subcomponent-level control or require extensive generative model training, limiting their practicality in low-data settings.

**Materials and methods:**

We propose the Brain Tumor Compositional Augmentation Pipeline (BT-CAP), a subcomponent-aware and anatomically constrained augmentation framework for multi-modal MRI. BT-CAP decomposes tumor subcomponents and recomposes them through a sequence of targeted operations, including isotropic scaling, B-spline deformation, Powell-optimized spatial rearrangement, inpainting, interface smoothing, and constrained edema deformation, applied consistently across all MRI modalities and segmentation masks, producing label-ready augmented volumes without additional annotation. We evaluated augmentation diversity (SSIM, label distribution, intensity variation, centroid displacement) and anatomical plausibility on 50 BraTS-PEDs 2025 cases, yielding 250 augmented volumes, and assessed segmentation performance on the full 256-case dataset using 3-fold cross-validation.

**Results:**

BT-CAP achieved a mean SSIM of 0.956 ± 0.014 with wide subcomponent volume change ranges and realistic intensity heterogeneity, confirming meaningful structural diversity. By architectural design, all augmented segmentation mask voxels were confined within brain boundaries, and zero overlap between deformed edema and the tumor core was observed across all 250 cases. Segmentation experiments showed mean Dice score improvements of 6%–7% for tumor subcomponents and 2%–3% for tumor core and whole tumor compared to training without compositional augmentation, with a computational cost of approximately 2 min per case on CPU.

**Conclusion:**

BT-CAP establishes a new class of compositional augmentation methods that deliver anatomically structured, label-ready, and scalable data generation without generative model training. The framework is applicable to any multi-class segmentation task where data scarcity and structural fidelity are critical, and is openly available at https://github.com/dramintavallaii/BT-CAP.

## 1 Introduction

Deep learning has achieved state-of-the-art performance in medical image segmentation; however, its success remains fundamentally limited by data scarcity, class imbalance, and the difficulty of obtaining high-quality expert annotations (1–3). In brain tumor MRI, these challenges are amplified by the heterogeneous composition of tumors, where subcomponents such as enhancing tumor (ET), non-enhancing tumor (NET), cystic component (CC), and peritumoral edema (ED) vary widely in size, spatial distribution, and prevalence across cases, particularly in pediatric populations where annotated datasets are small and inter-case variability is high (4, 5). Data augmentation is therefore essential to improve generalization and mitigate class imbalance, but existing strategies fail to address three critical requirements simultaneously: (1) independent manipulation of tumor subcomponents to enrich minority class representation, (2) enforcement of anatomical plausibility across modalities, and (3) production of label-ready augmented volumes without additional manual annotation effort (6–9).

Existing augmentation strategies for medical image segmentation can be broadly categorized into four approaches, each with characteristic limitations. Classical geometric and intensity augmentation (random flips, rotations, elastic deformations, brightness and contrast shifts) operates at the whole-image level and cannot alter the spatial distribution or relative composition of tumor subcomponents, leaving class imbalance among subregions unaddressed (7, 9). GAN-based synthesis (6, 8, 10–12) can produce high-fidelity synthetic images and has shown utility in augmenting liver lesion and brain tumor datasets; however, it requires GPU-intensive adversarial training, depends on sufficiently large training sets to prevent mode collapse, and frequently requires manual post-processing to verify label alignment in the synthesized output. Lesion-aware mixing approaches such as CarveMix (13) stochastically combine pairs of annotated images by pasting lesion-region crops between volumes, preserving label alignment and improving segmentation accuracy; however, these methods operate at the level of the whole lesion region and do not provide independent manipulation of individual tumor subcomponents or anatomically constrained deformation of surrounding tissue. Diffusion model-based synthesis (14, 15) has recently achieved impressive realism in brain MRI generation, conditioned on demographic covariates or anatomical priors, but similarly requires substantial compute for model training and does not directly address subcomponent-level label diversity. Synthesis-without-images approaches such as SynthSeg (16) generate training images from segmentation label maps alone, enabling contrast-agnostic segmentation; however, these are designed for whole-brain anatomy rather than intra-tumoral subcomponent augmentation. Across all five categories, no existing approach provides subcomponent-level spatial rearrangement with guaranteed label alignment, modality-consistent transformations, and no generative model training requirement.

To address these gaps, we introduce the Brain Tumor Compositional Augmentation Pipeline (BT-CAP), a subcomponent-aware and anatomically constrained augmentation framework for multi-modal MRI. BT-CAP decomposes each tumor into its constituent subcomponents (ET, NET, CC, ED) and recomposes them through a sequence of targeted operations, including isotropic scaling, B-spline deformation, Powell-optimized spatial rearrangement, inpainting, interface smoothing, and constrained edema deformation, applied consistently across all four MRI modalities and the segmentation mask. This design directly addresses the three limitations identified above: (1) subcomponent-level manipulation enriches minority class representation by independently rescaling, deforming, and repositioning each subregion; (2) anatomical constraints enforced at every step ensure all augmented voxels remain within brain boundaries and edema deformation stays outside the tumor core; and (3) all transformations are applied simultaneously to the images and their masks, producing label-ready outputs without additional annotation. Unlike GAN-based or diffusion-based approaches, BT-CAP requires no generative model training, runs on a standard CPU in approximately 2 min per case, and is immediately deployable in low-data regimes where generative approaches would themselves be data-starved.

## 2 Materials and methods

BT-CAP enhances multi-modal MRI data for brain tumor analysis by manipulating tumor subcomponents (ET, NET, CC, ED) across four MRI modalities (T1-weighted, T1-weighted with contrast, T2-weighted, and FLAIR). Using the co-registered multi-modal volume, BT-CAP performs sequential operations to generate realistic augmented data while preserving anatomical plausibility. All transformations in BT-CAP are applied consistently across the modalities and the segmentation mask, ensuring that the augmented volumes and their corresponding labels remain aligned, eliminating the need for additional manual labeling or segmentation. The following subsections outline each step, with detailed implementations available at the author's GitHub repository (https://github.com/dramintavallaii/BT-CAP).

### 2.1 Loading multi-modal MRI and segmentation data

This step initializes the BT-CAP by loading multi-modal MRI data and segmentation masks, ensuring spatial alignment through metadata extraction. It processes T1-weighted, T1-weighted with contrast, T2-weighted, and FLAIR images, along with their corresponding brain masks and a segmentation mask that defines tumor subcomponents. Metadata, including spatial transformations, is extracted to maintain co-registration for subsequent steps.

### 2.2 Core manipulation

#### 2.2.1 Extracting tumor subcomponents

This step isolates the ET, NET, and CC from the multi-modal MRI data and segmentation mask. Each subcomponent (ET, NET, CC) is extracted by computing its tight bounding box, cropping the corresponding region from all modalities and the segmentation mask, and generating a binary mask for the target label. For components exceeding 100 voxels, binary morphological opening with a 6-connectivity structuring element is applied to smooth the extracted mask and remove isolated noise voxels. Components with fewer than 100 voxels after opening are retained without smoothing to preserve small subcomponents. Modality intensities outside the binary mask are set to zero to isolate the subcomponent for downstream operations.

#### 2.2.2 Random scaling and reinsertion of tumor subcomponents

BT-CAP applies random isotropic scaling to each extracted tumor subcomponent (ET, NET, CC) to simulate variations in tumor size. For each subcomponent, a scale factor is drawn uniformly from [1.3, 1.5], representing an enlargement of 30%–50% relative to the original size. Modality intensities are resampled using cubic spline interpolation (order = 3) and subsequently clipped to the original per-modality intensity range within the subcomponent mask, preventing interpolation-induced intensity artefacts. The corresponding segmentation mask is resampled using nearest-neighbour interpolation (order = 0) to preserve discrete label values. After scaling, both the resampled modalities and mask are zeroed outside the binary subcomponent mask. The scaled subcomponent is then reinserted into the full-volume space, centered on the original bounding box centroid, using zero-padding to match the original image dimensions. This scaling is applied identically across all four MRI modalities and the segmentation mask to maintain label consistency.

#### 2.2.3 B-spline deformation and reinsertion of tumor subcomponents

Non-rigid deformation is applied to each rescaled tumor subcomponent using volumetric B-spline transformations to introduce realistic shape variability. Before deformation, each subcomponent is cropped to a tight bounding box with a 10-voxel margin to reduce computational overhead. B-spline control point grid spacing and displacement magnitude are adapted to the physical size of each subcomponent. Specifically, a scale factor *s* = (*V*/10,000)^(1/3)^ is computed from the subcomponent volume *V* in mm^3^, clamped to [0.5, 2.0], and used to modulate both the base grid spacing (10 mm) and base displacement magnitude (5 mm), yielding physically proportionate deformations for both small and large subcomponents. Random displacements are applied to B-spline control point parameters by sampling uniformly from [−3s, +3s] times the adaptive displacement magnitude. B-spline basis functions of order 3 were used for control point interpolation. Modality intensities were resampled with linear interpolation; the segmentation mask was resampled with nearest-neighbour interpolation. After deformation, binary morphological opening with a 6-connectivity structure is applied to the deformed mask to remove artefactual surface voxels. The deformed subcomponent is then reinserted into the full-volume space at the original bounding box location.

#### 2.2.4 Rearranging tumor subcomponents for optimal core coverage

This step orchestrates the spatial rearrangement of deformed tumor subcomponents within the original tumor core footprint, forming the central operation of BT-CAP. Each subcomponent volume is first decomposed into spatially disconnected islands using 6-connectivity labeling, sorted by size in descending order. Islands comprising fewer than 100 voxels are discarded. Large islands exceeding a splitting threshold *T* = max(100, |*C*|/2), where |*C*| is the tumor core volume in voxels, are recursively split into two sub-islands using watershed segmentation on the Euclidean distance transform, with peaks identified by distance-transform local maxima as seeding points. This splitting prevents any single subcomponent from dominating the core coverage.

Each island is then optimally placed within the tumor core using a 6-degree-of-freedom spatial transformation comprising three Euler rotation angles [sampled from (−180°, 180°)] and three translational shifts (bounded by ±50% of the tumor core bounding box dimensions). Optimization is performed using Powell's method (maximum 80 iterations, parameter tolerance xtol = 0.2, function tolerance ftol = 0.1), minimizing the negative fractional overlap between the transformed island and the uncovered portion of the tumor core. The optimizer is initialized with the translation set to the vector from the island center of mass to the center of mass of the currently uncovered core region. Islands are processed in descending size order across all subcomponent types (ET, NET, CC) simultaneously, so the largest island, regardless of label, is placed first. Rotations use a ZYX Euler convention (Rz @ Ry @ Rx); modality intensities are transformed using cubic spline interpolation (order = 3) and the mask with nearest-neighbour interpolation (order = 0). After placement, each transformed island mask is lightly smoothed with a Gaussian filter (*σ* = 1.0) and thresholded at 0.6 to reduce binary mask edge artefacts. Only the portion of the placed island overlapping with the original tumor core is retained. The final composition layers NET → CC → ET, such that ET voxels take priority in any overlap regions. Interface smoothing (see Section 2.3.2) is applied after each layer has been composited. The formal algorithm is described in Algorithm 1.

Algorithm 1 **Subcomponent Rearrangement into Tumor Core**

Input:

  Per-label deformed pairs {(*M*_*ℓ*, *V*_*ℓ*)} for *ℓ* ∈ {1 = ET, 2 = NET, 3 = CC}

  Original tumor core mask C; random number generator rng

Output:

  Composed segmentation mask M_out; composed modality volume V_out

▶ *Island preparation*

1. For each label *ℓ*:

    Extract connected components of *M*_*ℓ*; sort by size (largest first)

    For each component *I* with |*I*| ≥ 100 voxels:

      If |*I*| > *T* = max(100, |*C*|/2):

       Recursively split I via watershed(distance_transform_edt(*I*),

       markers = 2 local maxima)

       Retain sub-islands with |sub| ≥ 100 voxels

    Append (|*I*|, *I*, *ℓ*, *V*_*ℓ*) to list L_*ℓ*

2. Merge L_ET ∪ L_NET ∪ L_CC → global list L

  Sort *L* by size, largest first

▶ *Greedy placement loop*

3. Initialize: covered_core ← ∅

  For each *ℓ*: placed_mask_*ℓ* ← 0; placed_vol_*ℓ* ← 0

4. For each (size, island *I*, label *ℓ*, source *V*_*ℓ*) in *L*:

  empty_core ← *C*\covered_core

  init_shift ← clip(COM(empty_core) − COM(I), ±50% × bbox(C))

  init_angles ← rng.uniform(−45°, +45°, size = 3) ▷ initialization only; Powell explores unbounded

  *x*_0_ ← [init_angles; init_shift]

  Define cost(*θ*):

    *Ĩ* ← affine_transform(*I*, *R*(*θ*[:3]), offset(*θ*[3:]), order = 0)

    If |*Ĩ*| = 0: return 1 × 10^6^

    return −|*Ĩ* ∩ empty_core|/|*Ĩ*|

  θ* ← Powell(cost, *x*_0_, maxiter = 80, xtol = 0.2, ftol = 0.1)

  *Ĩ* ← affine_transform(*I*, *R*(θ*[:3]), offset(θ*[3:]), order = 0)

  *Ṽ* ← affine_transform(*V*_*ℓ*, R(θ*[:3]), offset(θ*[3:]), order = 3) × *Ĩ*

  *Ĩ* ← (gaussian_filter(*Ĩ*, *σ* = 1.0) > 0.6) ▷ edge smoothing

  within ← *Ĩ* ∩ *C* ▷ clip to core

  placed_vol_*ℓ*[:, within] ← *Ṽ*[:, within]

  placed_mask_*ℓ*[within] ← *ℓ*

  covered_core ← covered_core ∪ within

▶ *Post-processing per label*

5. For each label *ℓ*:

  Detect outlier voxels: 0.6745 × |*x* − median|/MAD > 3.5

  Replace outliers with k-NN mean (*k* = 5, KD-tree on valid voxels)

  Find single enclosed empty voxels (all 6 face-neighbours filled)

  Fill enclosed voxels by face-neighbour mean

▶ *Composition (ET takes priority over CC over NET)*

6. *M*_out ← 0; *V*_out ← 0

  For *ℓ* in [2 (NET), 3 (CC), 1 (ET)]:

   V_out[:, placed_mask_*ℓ* > 0] ← placed_vol_*ℓ*[:, placed_mask_*ℓ* > 0]

   Apply interface smoothing at the layer boundary

   (*α* = 0.7, kernel 3 × 3 × 3, 2 dilation iterations)

   M_out[placed_mask_*ℓ* > 0] ← *ℓ*

Return M_out, V_out

*Notation: C* *=* *original tumor core mask; |·|* *=* *voxel count; COM* = *center of mass; bbox* = *bounding box; R(θ)* *=* *ZYX Euler rotation matrix; affine_transform uses order=0 (mask) or order=3 (modalities); gaussian_filter with σ* *=* *1.0.*

### 2.3 Refinement

#### 2.3.1 Inpainting empty areas in the tumor core

Following the rearrangement, voxels within the original tumor core footprint that are not assigned to any subcomponent during the placement step are identified as empty areas. These gaps arise when the transformed islands do not fully tile the core, a natural consequence of shape-constrained placement. Connected components of empty voxels within the core are identified using 6-connectivity labeling. For each empty component, a donor subcomponent label is selected as follows: components of 10 voxels or fewer are assigned the label of the most frequently occurring neighbor in the adjacent filled region; larger components are assigned NET (label 2) by default, falling back to CC (label 3) or ET (label 1) if NET is absent. For each MRI modality channel, fill intensities are sampled from a Gaussian distribution *N*(*µ*, *σ*), where *µ* and *σ* are the mean and standard deviation of the donor label's intensities in the current augmented volume, introducing realistic stochastic heterogeneity consistent with the donor tissue. The sampled values are placed into the empty region and then smoothed using a Gaussian filter (*σ* = 1.0) applied within a locally padded subvolume (padding = 3 × *σ* = 3 voxels) to avoid edge effects. The corresponding segmentation mask voxels are assigned the donor label. These operations are applied identically across all four MRI modalities.

#### 2.3.2 Smoothing interfaces and managing outlier intensities

Interface smoothing and outlier intensity correction are applied throughout the pipeline at boundaries between subcomponents and between the tumor and surrounding brain tissue. Interface smoothing operates by first identifying the interface zone as the intersection of one region with the morphological dilation (2–3 iterations) of the adjacent region. At each voxel in the interface zone, the output intensity is computed as a weighted blend: I_out = (1 − *α*) × I_orig + *α* × I_local, where I_local is the mean intensity of foreground voxels within a local (3 × 3 × 3)-voxel neighborhood, and the blending coefficient *α* = 0.7. Outlier intensity correction is applied within each placed subcomponent using a modified *Z*-score criterion: voxels satisfying 0.6745 × |*x* − median|/MAD > 3.5 are flagged as outliers. Flagged voxels are replaced by the mean intensity of their *k* = 5 nearest non-outlier neighbors within the same subcomponent, identified using a KD-tree. A final global interface smoothing pass is applied at the boundary between the full augmented tumor region and the surrounding brain parenchyma, using a (2 × 2 × 2) kernel and *α* = 0.7 with 3 dilation iterations, ensuring a smooth transition that eliminates visible seam artefacts at the tumor-brain boundary.

### 2.4 Context adaptation

#### 2.4.1 Deforming peritumoral edema with anatomical constraints

Peritumoral edema (label 4) is deformed independently of the tumor core to simulate natural variability in edema extent and shape. A smooth random displacement field is generated as follows: a low-resolution random vector field is sampled from *N*(0, *m*) at a grid spacing of 16 voxels in each dimension, where the displacement magnitude m is drawn uniformly from [6, 12] mm per case. This low-resolution field is upsampled to full image resolution using cubic spline interpolation (order = 3) and then spatially smoothed with a Gaussian filter (*σ* = 5 voxels) to ensure field continuity and physiological plausibility. To preserve the integrity of the tumor core, displacement vectors are set to zero within the tumor core and deep edema regions (i.e., all edema voxels except a 5-voxel outer shell), enforcing that deformations act exclusively outward at the edema periphery. The resulting field is applied to edema voxels using map_coordinates with cubic interpolation; a binary threshold of 0.4 is applied to the warped edema indicator to obtain the deformed edema mask. Following deformation, the original tumor core intensities are restored at their original locations, ensuring that edema deformation does not alter the tumor core's spatial structure. Interface smoothing (*α* = 0.7, kernel 3 × 3 × 3, 2 dilation iterations) is applied at the boundary between the deformed edema and the frozen inner edema, and at the boundary between the tumor core and the surrounding edema, to eliminate abrupt intensity transitions.

#### 2.4.2 Normalizing and saving augmented MRI data

This final step in BT-CAP completes the pipeline by normalizing the augmented MRI modalities and segmentation mask to ensure consistency. Then it saves them in a standard format for downstream use. Each modality is normalized to standardize intensity ranges, facilitating compatibility with segmentation models. The segmentation mask, encompassing the augmented tumor subcomponents and edema, is constrained to the brain region using a consensus of brain masks across all modalities to ensure anatomical fidelity. Transformations are applied uniformly to maintain alignment between modalities and the mask, eliminating the need for manual labeling. The normalized modalities and mask are then saved as NIfTI files, preserving spatial metadata to maintain co-registration with the original BraTS dataset. This step ensures the augmented data are readily usable for training robust segmentation models, with all outputs organized for easy access and reproducibility.

### 2.5 Evaluation strategy

BT-CAP was evaluated in two complementary setups:

1. *Augmentation Fidelity and Diversity:* We applied BT-CAP to a subset of the Brain Tumor Segmentation (BraTS) Lighthouse Challenge 2025 Task 6 dataset, comprising 50 pediatric glioma cases, each with four MRI modalities (T1-weighted, T1-weighted with contrast, T2-weighted, and T2-FLAIR) and expert-annotated segmentation masks, generating 250 augmented volumes (5 per case). These were analyzed using SSIM, label distribution, intensity variation, and centroid displacement to assess diversity, and brain mask overlap/edema-core separation to ensure anatomical fidelity.
2. *Impact on Segmentation Performance:* To rigorously evaluate BT-CAP's contribution to segmentation accuracy, we used the full BraTS-PEDs 2025 training dataset (256 cases). Data were organized into a 3-fold cross-validation with 170 training + 86 validation cases per fold. The pipeline was applied only to the training data to prevent data leakage, resulting in 340 training volumes (170 original and 170 augmented) per fold. Validation sets remained untouched. An attention-guided 3D U-Net with multi-modal and channel-wise attention mechanisms was trained on the data.

### 2.6 Implementation details

BT-CAP was implemented in Python 3.10 using SimpleITK (version 2.4) for image I/O and spatial transformations, NumPy, SciPy, scikit-image, and scikit-learn for array operations, morphological processing, and nearest-neighbor search. Parallel processing across cases was managed using Python's concurrent.futures.ProcessPoolExecutor, with the number of workers adaptively limited to 75% of available system RAM divided by per-case memory estimate to prevent memory overflow. Reproducibility was ensured by deriving a per-case, per-sample random seed deterministically from the global base seed, case name (SHA-256 hash), and sample index via numpy.random.SeedSequence, such that all augmentation results are exactly reproducible given the same base seed. The complete source code is available at https://github.com/dramintavallaii/BT-CAP under an MIT License.

The segmentation experiments were conducted on a workstation with an NVIDIA RTX 3090 GPU (24 GB VRAM) and 64 GB RAM. The attention-guided 3D U-Net was implemented in PyTorch (version 2.3) and trained for 300 epochs per fold using the Adam optimizer (learning rate = 1 × 10^−4^) with a patch-based loading strategy (4 patches per volume). Conventional on-the-fly augmentation (±15° rotation, 50% random flip, ±10% intensity shift) was applied per epoch during training. The three cross-validation folds were formed by randomly partitioning the 256-case BraTS-PEDs 2025 training set with a fixed random seed, yielding 170 training and 86 validation cases per fold. Augmentation was applied exclusively to the training split of each fold to prevent data leakage; validation sets remained unmodified throughout. BT-CAP augmentation was run offline on an Intel Core i9-13900K, 24-core CPU, generating augmented volumes prior to training.

## 3 Results

### 3.1 Augmentation diversity

**Figure 1:**
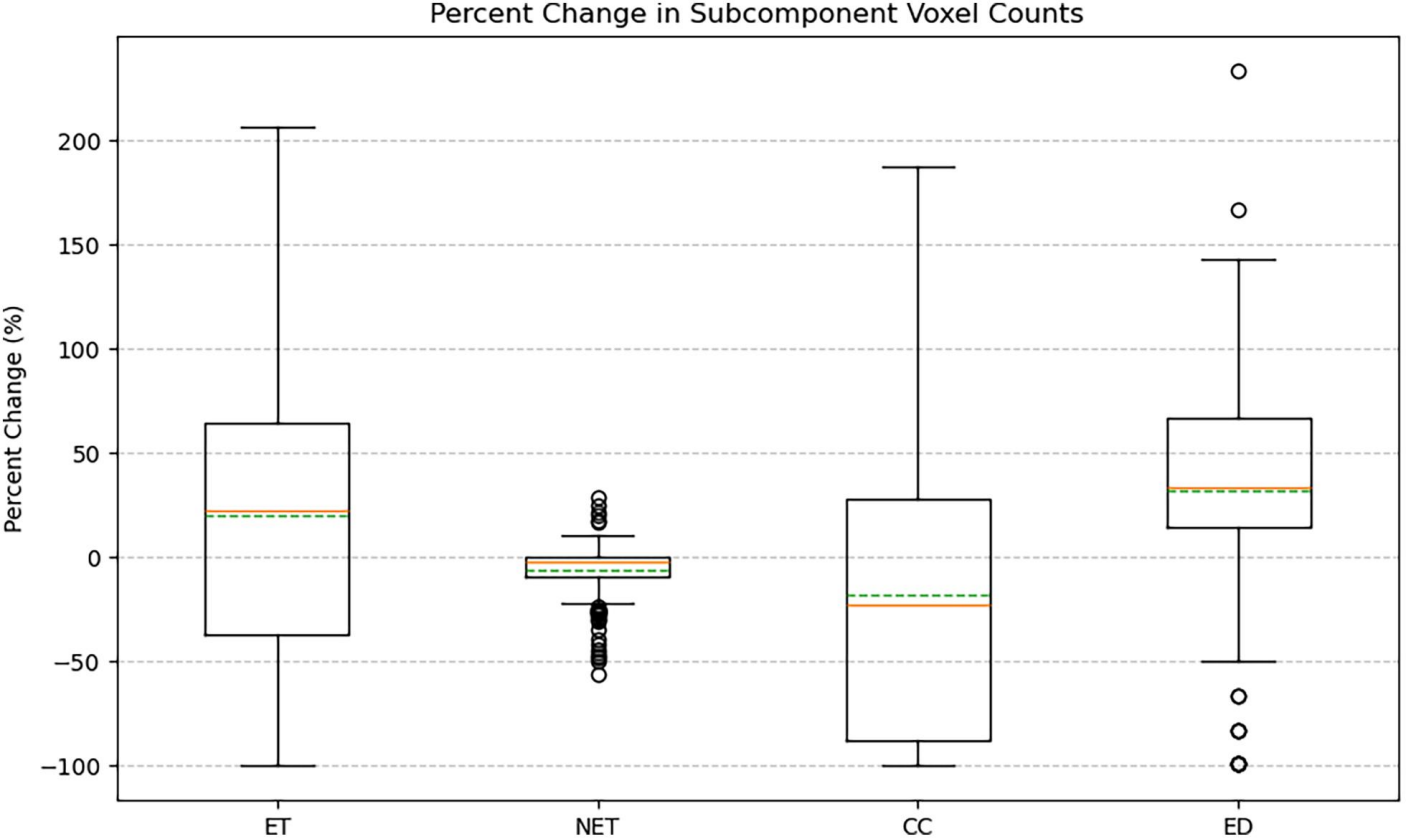
Label distribution changes across augmented pediatric glioma cases. Box plots showing the percent volume change for ET, NET, CC, and ED after augmentation. Wide ranges and numerous outliers reflect substantial diversity introduced by the pipeline, particularly for sparse subcomponents such as ET and CC.

**Figure 2:**
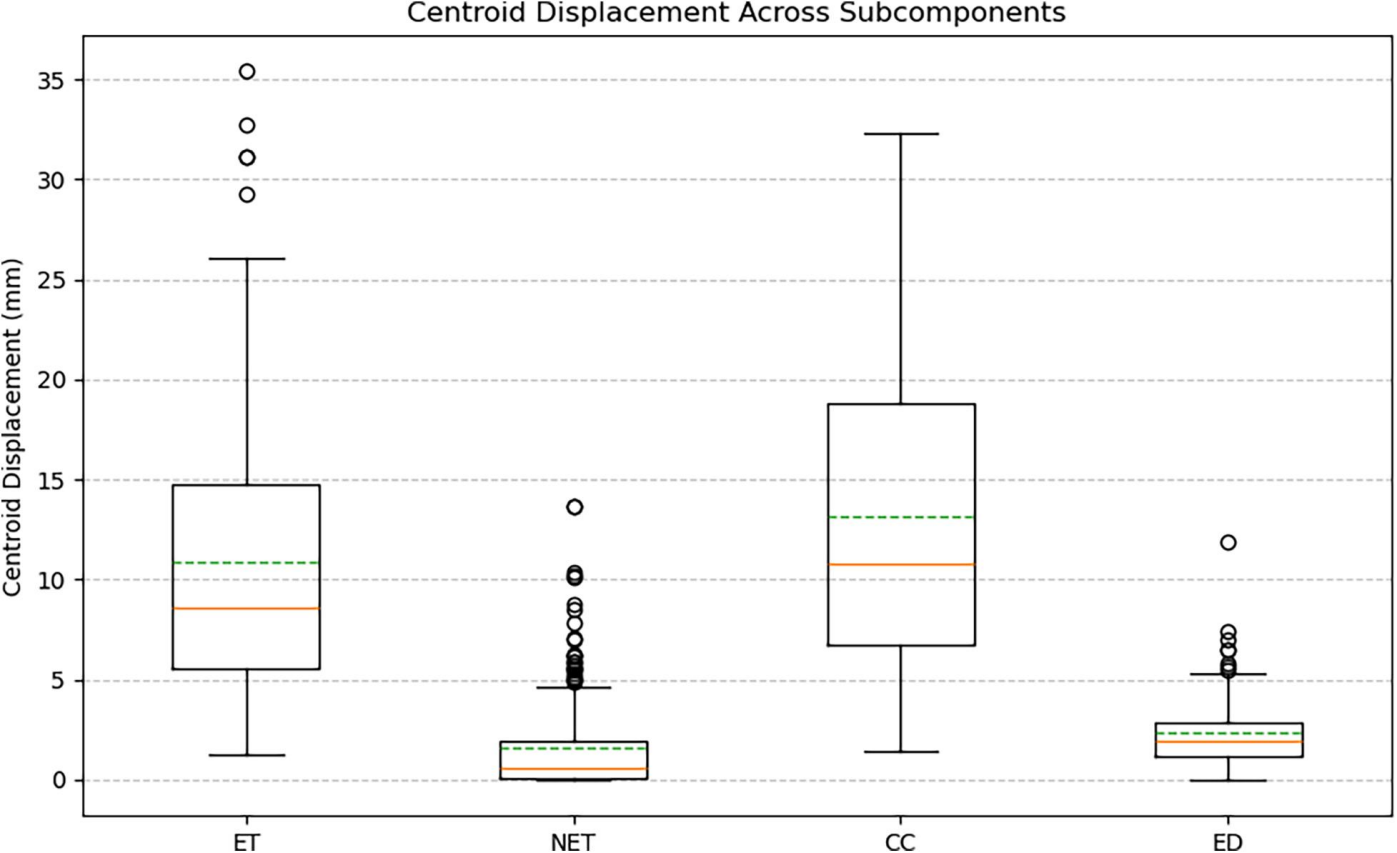
Intensity variation in augmented tumor subcomponents across MRI modalities. Box plots of the standard deviation of normalized intensities for ET, NET, CC, and ED in T1, T1-contrast, T2, and T2-FLAIR sequences. Variability patterns demonstrate modality-specific heterogeneity and realistic tissue simulation achieved by the inpainting and smoothing steps.

**Figure 3:**
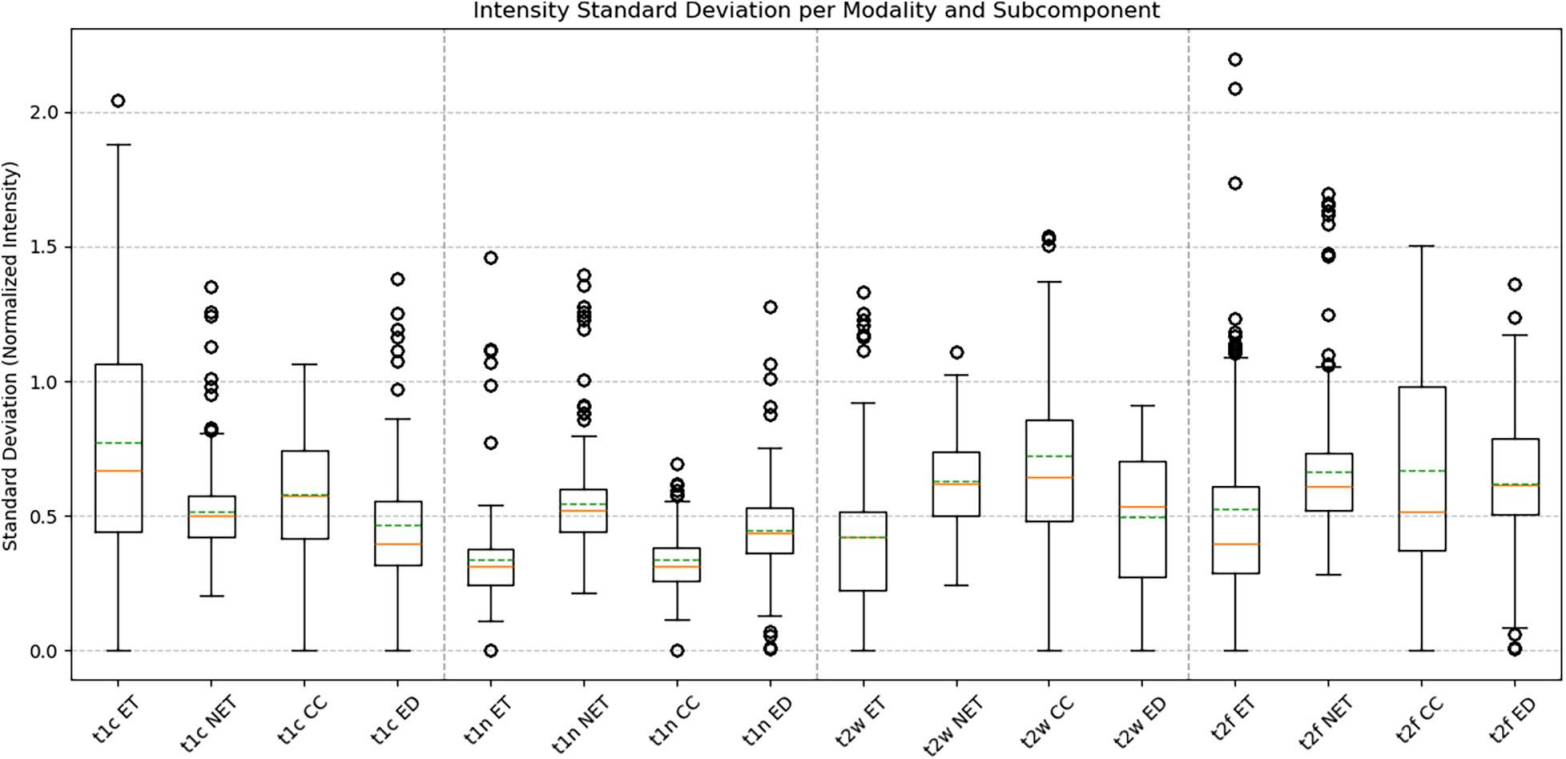
Spatial displacement of tumor subcomponents in augmented data. Box plots of centroid displacement (in millimeters) for ET, NET, CC, and ED between original and augmented volumes. Larger displacements for ET and CC indicate enhanced spatial diversity, while smaller shifts for NET and ED preserve anatomical plausibility.

Augmented volumes generated by BT-CAP maintained high structural fidelity, with a mean SSIM of 0.956 ± 0.014 across modalities, indicating high fidelity. Label distribution analysis revealed diverse redistributions: ET volume change in percent ranged from −99.75 to 206.19 (mean: 19.74 ± 71.09), NET from −56.11 to 29.04 (mean: −6.04 ± 11.70), CC from −99.73 to 187.02 (mean: −18.16 ± 73.53), and edema from −99.02 to 233.33 (mean: 31.76 ± 59.35), with wide interquartile ranges and outliers for more heavily augmented labels (ET, CC, ED). Figure 1's box plots illustrate this diversity, showing wide ranges and numerous outliers driven by the sparsity of pediatric glioma data, where small subcomponent volumes yield extreme percent changes. Intensity variations were realistic, with standard deviations of normalized intensities summarized in Table 1, reflecting modality-specific heterogeneity. Figure 2's box plots highlight the diverse intensity profiles across subcomponents and modalities, demonstrating the pipeline's robust tissue simulation capabilities. Centroid displacements averaged 10.84 ± 7.07 mm for ET, 1.56 ± 2.31 mm for NET, 13.13 ± 8.25 mm for CC, and 2.39 ± 2.00 mm for ED, reflecting spatial diversity (Figure 3).

**Table 1:** Intensity standard deviation ranges per modality and subcomponent.

Modality	ET std range	NET std range	CC std range	ED std range
T1	0–1.462	0.215–1.394	0–0.694	0.007–1.279
T1c	0–2.044	0.206–1.350	0–1.064	0.002–1.380
T2	0–1.331	0.246–1.110	0–1.538	0.002–0.911
FLAIR	0–2.199	0.284–1.695	0–1.502	0.007–1.359

### 3.2 Anatomical plausibility

Anatomical plausibility in BT-CAP is enforced by two hard architectural constraints built into the pipeline. First, all segmentation mask voxels are constrained to the brain region via an element-wise multiplication with the provided brain mask during the final processing step. This constraint held for 100% of voxels across all 250 augmented cases, confirming that no augmented subcomponent label was placed outside the anatomical brain boundary. Second, the edema displacement field is explicitly set to zero within the tumor core and inner edema regions, restricting deformations to the edema periphery and preventing inward distortion toward the core. Consequently, zero overlap between the deformed edema mask and the tumor core was observed across all 250 cases. These outcomes reflect the deterministic enforcement of design constraints rather than probabilistic outcomes, and hold by construction regardless of tumor morphology or case-specific variability.

**Figure 4:**
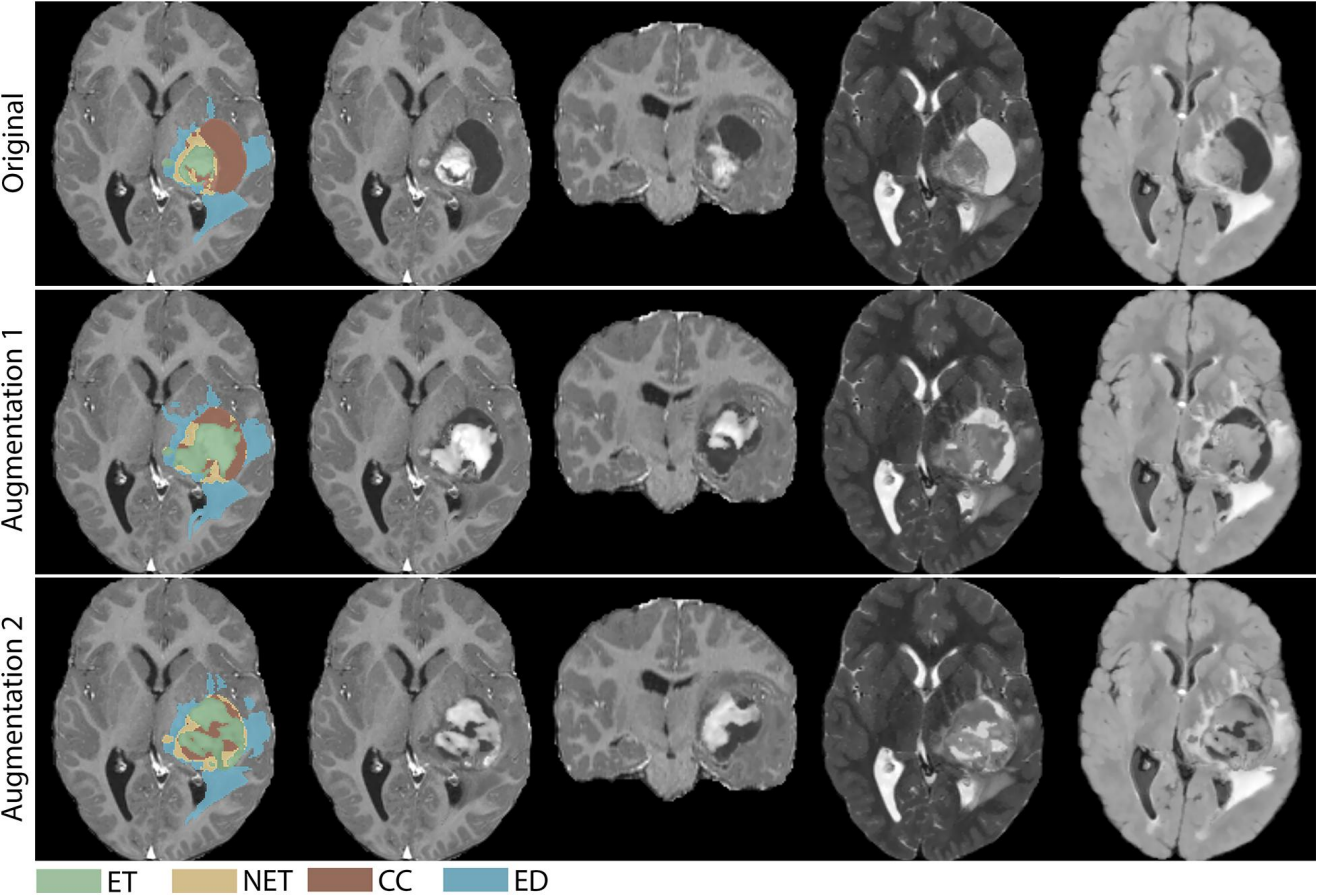
Qualitative examples of augmented multi-modal MRI volumes with corresponding segmentation masks. Representative axial and coronal slices from T1-contrast, and axial slices from T2, and T2-FLAIR sequences of the original image and two augmentations, alongside the updated segmentation masks. The augmented cases demonstrate realistic tumor morphologies, smooth subcomponent interfaces, anatomically constrained edema deformation, and absence of artifacts, highlighting the pipeline's ability to maintain fidelity while introducing substantial variability.

Qualitative inspection of representative axial and coronal slices (Figure 4) confirmed that these constraints produced visually coherent results across augmented cases. Subcomponent interfaces were smooth, edema deformation was spatially plausible and confined to the tumor periphery, and no visible seam artefacts or implausible intensity discontinuities were present. Full independent anatomical plausibility validation, such as expert radiologist rating or quantitative topology metrics, was not performed in this study and is identified as a primary direction for future work.

### 3.3 Segmentation performance

The augmentation pipeline significantly enhanced segmentation performance on the full BraTS-PEDs 2025 dataset, addressing its limited size (256 training cases) and multi-institutional variability (4). Conventional augmentation (e.g., ±15° rotations, 50% flips, ±10% intensity shifts) was applied dynamically per epoch, with patch-based loading (4 patches/volume) and 300-epoch training (36 h/fold). An ablation study (Table 2) validated BT-CAP's contribution to segmentation performance across both Dice score and Hausdorff Distance 95th percentile (HD95). Removing the compositional augmentation pipeline reduced mean Dice scores by 6%–7% across the four tumor subcomponents (ET: 0.81 ± 0.01 vs. 0.75 ± 0.02; NET: 0.87 ± 0.01 vs. 0.80 ± 0.01; CC: 0.77 ± 0.02 vs. 0.71 ± 0.02; ED: 0.95 ± 0.01 vs. 0.89 ± 0.01), and by 4% and 2% for TC and WT, respectively. HD95 was correspondingly higher without augmentation, with the largest absolute increases in ET (9.2 ± 1.8 vs. 15.3 ± 2.4 mm) and CC (12.4 ± 2.3 vs. 19.7 ± 3.1 mm), reflecting the reduced boundary precision for the sparsest subcomponents. Improvements were consistent in direction across all three cross-validation folds. These gains are particularly notable for the sparsest and most clinically significant subcomponents (ET, CC), where the rearrangement and inpainting steps most directly increase training sample diversity. We acknowledge that with three folds, the sample size is insufficient for formal significance testing; the reported per-fold consistency provides practical evidence of BT-CAP's contribution within these experimental constraints.

**Table 2:** Mean ± SD dice scores and HD95 (mm) from the ablation study on the BraTS-PEDs 2025 dataset, comparing training with and without pre-training application of the compositional augmentation pipeline. Values represent means and standard deviations across three cross-validation folds. HD95 is reported in millimeters; lower values indicate better boundary delineation.

Configuration	ET	NET	CC	ED	TC	WT
With BT-CAP (Dice)	0.81 ± 0.01	0.87 ± 0.01	0.77 ± 0.02	0.95 ± 0.01	0.91 ± 0.01	0.90 ± 0.01
Without BT-CAP (Dice)	0.75 ± 0.02	0.80 ± 0.01	0.71 ± 0.02	0.89 ± 0.01	0.87 ± 0.01	0.88 ± 0.01
With BT-CAP (HD95)	9.2 ± 1.8	7.1 ± 1.2	12.4 ± 2.3	11.3 ± 1.9	8.2 ± 1.5	10.6 ± 1.7
Without BT-CAP (HD95)	15.3 ± 2.4	11.8 ± 1.9	19.7 ± 3.1	16.2 ± 2.2	13.1 ± 2.0	14.4 ± 1.8

### 3.4 Computational efficiency

BT-CAP processed each subject in approximately 2 min on a standard CPU, generating five augmented volumes per case. This efficiency supports scalable applications to large datasets, contrasting with GAN-based methods, which often require GPU-intensive training and post-processing.

## 4 Discussion

The Brain Tumor Compositional Augmentation Pipeline (BT-CAP) presented in this study addresses two persistent challenges in medical image segmentation: data scarcity and class imbalance among tumor subcomponents. By manipulating tumor subcomponents and peritumoral edema through a multi-step compositional process, including extraction, scaling, B-spline deformation, Powell-optimized rearrangement, inpainting, interface smoothing, and constrained edema deformation, BT-CAP generates diverse, anatomically structured augmented volumes with guaranteed label alignment. Unlike GAN-based augmentation (8, 10–12) and diffusion model-based synthesis (14, 15), which require GPU-intensive training, depend on large training sets, and may not guarantee label correctness, BT-CAP is training-free, runs entirely on CPU in approximately 2 min per case, and produces label-ready augmented volumes by architectural construction. This positions BT-CAP as a uniquely practical complement to existing synthesis approaches.

Our evaluation of 50 BraTS-PEDs pediatric glioma cases, generating 250 augmented volumes, demonstrated meaningful diversity across all measured dimensions. High structural similarity (SSIM: 0.956 ± 0.014) confirmed that augmented volumes remained anatomically coherent with their source cases rather than introducing implausible global distortions. Wide label volume change ranges (ET: −99.75% to +206.19%, CC: −99.73% to +187.02%, ED: −99.02% to +233.33%) reflect the substantial spatial rearrangement introduced by the compositional pipeline, particularly for sparse subcomponents where small absolute volumes translate to large percent changes. Intensity variation across modalities (Table 1) further demonstrated that the inpainting and smoothing steps produce realistic tissue heterogeneity consistent with the expected modality-specific appearance of each subcomponent. Centroid displacements (ET: 10.84 ± 7.07 mm, CC: 13.13 ± 8.25 mm) confirmed spatial diversity in the most heavily rearranged subcomponents, while the smaller displacements in NET (1.56 ± 2.31 mm) and ED (2.39 ± 2.00 mm) reflect their anatomical anchoring within the original core footprint (Figures 1–3). We note that the high mean SSIM also implies a degree of structural conservatism, which is expected given that the pipeline preserves brain parenchyma and applies transformations only within the tumor region. This is a deliberate design choice, not a limitation, as it ensures that background anatomy remains unaltered and training signals remain clinically meaningful.

Anatomical plausibility in BT-CAP is ensured by design through two hard constraints embedded in the pipeline architecture. First, all tumor subcomponent voxels are masked to the brain region via a final element-wise multiplication with the brain mask during post-processing, yielding 100% containment of segmentation mask voxels within the brain boundary across all 250 augmented cases. Second, the edema deformation field is set to zero within the tumor core and inner edema regions, enforcing that deformations act exclusively at the edema periphery and guaranteeing zero overlap between the deformed edema and the tumor core. These are architectural guarantees rather than independently discovered empirical outcomes, and their value lies in the fact that they hold by construction across all inputs regardless of tumor morphology or case-specific variability. Qualitative inspection of 2D axial and coronal slices (Figure 4) confirmed that these constraints produced visually coherent results: subcomponent interfaces are smooth, edema deformation is spatially plausible, and no visible seam artefacts or implausible intensity discontinuities are present. We acknowledge that full independent anatomical plausibility validation, for example, expert radiologist rating or quantitative topology metrics, would provide additional clinically grounded evidence beyond what computational metrics can supply, and we identify this as a primary direction for future work.

The ablation study (Table 2) validated BT-CAP's contribution to segmentation performance. Removing the compositional augmentation pipeline reduced mean Dice scores by 6%–7% across the four tumor subcomponents (ET, NET, CC, ED) and by 4% and 2% for the tumor core and whole tumor, respectively, consistent across all three cross-validation folds. These gains are particularly notable for the sparsest and most clinically significant subcomponents (ET, CC), where the rearrangement and inpainting steps most directly increase training sample diversity. We acknowledge that with three cross-validation folds, the statistical power to formally distinguish these improvements from fold-to-fold variability is limited; future large-scale experiments should include significance testing across a greater number of folds or independent test sets. The consistency of improvements across all six targets and all three folds, however, provides practical evidence of BT-CAP's contribution beyond what would be expected from chance variation. The computational overhead of BT-CAP is negligible relative to model training, making it practical for large-scale deployment without additional GPU resources.

### 4.1 Comparison with existing augmentation strategies

BT-CAP occupies a distinct and complementary position relative to existing data augmentation approaches. Classical geometric augmentation methods, including random rotations, flips, elastic deformations, and affine transforms, operate at the whole-image level and cannot alter the spatial distribution or relative arrangement of tumor subcomponents within a scan. These methods are useful for improving rotational and positional invariance but do not address class imbalance among subcomponents or introduce structural diversity in tumor morphology. In our experiments, conventional geometric and intensity augmentation (±15° rotation, 50% flip, ±10% intensity shift) was applied on-the-fly during training in addition to BT-CAP's offline compositional augmentation, confirming that the two approaches are complementary rather than competing. Intensity augmentation methods, such as brightness and contrast shifts, noise injection, and histogram matching, similarly operate globally and cannot introduce subcomponent-level morphological variation. Among lesion-aware mixing approaches, CarveMix (13) represents the closest published method to BT-CAP's compositional philosophy, combining pairs of annotated brain lesion images by carving and pasting lesion-region crops, with mass-effect modeling for brain tumors specifically. BT-CAP substantially extends this paradigm by decomposing tumors into individual subcomponent types (ET, NET, CC, ED), applying independent scaling and B-spline deformation per subcomponent, optimizing spatial rearrangement within the tumor core through a 6-DOF Powell optimization, and separately constraining edema deformation, capabilities none of which CarveMix addresses. GAN-based augmentation (8, 10–12) and diffusion model-based synthesis (14, 15) can produce high-fidelity synthetic images but require substantial GPU training (hours to days), depend on datasets large enough to train the generative model, may not guarantee the correctness of segmentation labels in the synthesized output, and introduce a second training problem with its own hyperparameter optimization. BT-CAP requires no training whatsoever, runs on a standard CPU, processes each case in approximately 2 min, and produces fully label-aligned outputs by construction. To our knowledge, BT-CAP is the only augmentation approach that provides subcomponent-level spatial rearrangement with guaranteed anatomical boundary enforcement, modality-consistent transformations, and no generative model dependency, making it directly usable in low-data regimes where GAN or diffusion-based approaches would themselves be data-starved.

### 4.2 Generalizability

Beyond pediatric brain tumor MRI, BT-CAP's design is inherently dataset-agnostic. The pipeline requires no dataset-specific tuning, no learned parameters, and no data-driven priors. Its only dataset-specific input is the segmentation label convention, which can be trivially remapped for any multi-class segmentation task. This makes BT-CAP directly applicable to adult glioma datasets [e.g., BraTS 2024 Adult (17)], meningioma cohorts, brain metastases, and multi-class segmentation problems in other organ systems. The same compositional principles, like extract subcomponents, rearrange within a boundary constraint, and recompose with smoothed interfaces, transfer directly to liver lesion segmentation (where tumor, necrosis, and enhancement zones correspond to ET, NET, and CC), cardiac substructure delineation, and musculoskeletal pathology. By framing augmentation as a compositional problem with anatomical constraints, BT-CAP introduces a methodological paradigm that is not inherently domain-specific. We acknowledge that this generalizability argument is currently theoretical: the present study validates BT-CAP exclusively on the BraTS-PEDs 2025 pediatric glioma dataset (4), and empirical cross-dataset validation remains future work. A concrete roadmap for this validation includes: (1) adult glioma using BraTS 2024, which shares the same label convention and would directly test generalization across age group and tumor grade; (2) multi-institutional datasets with deliberate domain shift, to test robustness to scanner variability; and (3) non-brain organ applications, beginning with liver lesion segmentation given its analogous multi-class label structure. The open-source release of BT-CAP is intended to facilitate this independent validation by the broader community.

### 4.3 Limitations

Several limitations of the current study should be noted. First, validation was conducted on a single dataset [BraTS-PEDs 2025, 256 cases (4)], limiting the generalizability of the quantitative findings to other tumor types, imaging protocols, and patient populations. Second, the scaling factor range [1.3, 1.5] and the displacement magnitude range [6, 12] mm were set empirically based on the characteristics of the BraTS-PEDs dataset; these may require adjustment for datasets with different tumor size distributions. Third, the ablation study used three cross-validation folds, which provides limited statistical power for formal significance testing, and future work should replicate these experiments with a larger number of cases and folds. Fourth, anatomical plausibility was assessed through a combination of design-guaranteed constraints and qualitative inspection rather than independent expert radiologist rating; while the former is rigorous by construction, and the latter is consistent with practice in the augmentation literature (10, 11, 13), a quantitative expert-grounded plausibility scoring would provide stronger clinical validation. Fifth, BT-CAP operates exclusively on the tumor region and does not model augmentation of the surrounding healthy brain parenchyma; for tasks where peritumoral tissue appearance is diagnostically relevant, complementary whole-brain augmentation strategies may be needed. Finally, the rearrangement step uses Powell's method with fixed iteration limits and tolerance parameters; for very large or geometrically complex tumors, additional iterations or a multi-start strategy may improve placement coverage.

Clinically, BT-CAP holds direct potential for improving segmentation accuracy in data-scarce scenarios, particularly relevant in pediatric oncology, where annotated MRI datasets are substantially smaller than adult tumor cohorts due to lower disease incidence and the ethical and logistical challenges of large-scale pediatric data collection. Improved segmentation accuracy translates directly to more reliable volumetric response assessment, treatment planning, and radiation therapy targeting. The open-source implementation under an MIT License promotes reproducibility, independent validation, and clinical translation, positioning BT-CAP as a practical tool for both research groups developing segmentation models and clinical sites seeking to augment small local datasets. Future integration with automated neural architecture search or end-to-end trainable augmentation policies could further optimize the pipeline's hyperparameters adaptively during training, reducing the need for manual parameter selection and extending applicability across diverse imaging contexts.

## 5 Conclusion

In this paper, we presented the Brain Tumor Compositional Augmentation Pipeline (BT-CAP), a subcomponent-aware and anatomically constrained framework for augmenting multi-modal brain tumor MRI data. By addressing the limitations of traditional geometric augmentation, GAN-based synthesis, and diffusion model-based synthesis, BT-CAP represents a methodological step forward in medical image analysis that delivers subcomponent-level structural diversity, guaranteed label alignment, and anatomical boundary enforcement without requiring any generative model training.

Beyond its immediate application to pediatric brain tumor MRI, BT-CAP offers a generalizable paradigm for compositional augmentation. The core principles, including subcomponent-aware manipulation, anatomically constrained deformation, and modality-consistent transformations, can be extended to other multi-class medical imaging domains where subcomponent imbalance and structural fidelity are critical, including liver lesion segmentation, cardiac substructure delineation, and musculoskeletal pathology.

Looking forward, future work will focus on validating BT-CAP across adult glioma and non-glioma tumor cohorts, extending it to multi-institutional datasets with scanner variability, and exploring integration with end-to-end trainable augmentation frameworks. Expert radiologist evaluation of augmented data quality and formal cross-dataset benchmarking remain important next steps toward broader clinical adoption.

In conclusion, BT-CAP delivers a computationally efficient, anatomically faithful, and openly available augmentation framework that directly strengthens deep learning-based segmentation. By bridging the gap between data scarcity and model performance without the overhead of generative model training, it provides a scalable and immediately deployable pathway toward more robust and clinically useful medical image analysis systems.

## Data Availability

All data, code, and implementations used in this study are openly available at the following GitHub repository: https://github.com/dramintavallaii/BT-CAP. Further inquiries can be directed to the corresponding author.
